# The role and future of the population health observatory: advancing public health intelligence in Saudi Arabia

**DOI:** 10.3389/fpubh.2026.1758899

**Published:** 2026-02-09

**Authors:** Mariam M. Al Eissa, Malak E. Aloufi, Abdulaziz Eskandarani, Aisha M. Alshehri, Mohammed M. Alrwois, Yahya A. AlMazni, Shaker A. Alomary, Abdullah Assiri, Mohammed AlAbdulaali

**Affiliations:** 1 College of Medicine, Alfaisal University, Riyadh, Saudi Arabia; 2 Population Health Observatory, Ministry of Health, Riyadh, Saudi Arabia; 3 Public Health Lab, Public Health Authority, Riyadh, Saudi Arabia; 4 King Khaled Eye Specialist Hospital (KKESH) Research Centre, Riyadh, Saudi Arabia; 5 Computational Sciences Department at the Centre for Genomic Medicine (CGM), King Faisal Specialist Hospital and Research Center, Riyadh, Saudi Arabia

**Keywords:** population health observatory, public health intelligence, health equity, AI in healthcare, vision 2030

## Abstract

The Population Health Observatory (PHO), established by Saudi Arabia’s Ministry of Health, employs cutting-edge technology, machine learning (ML), and proactive forecasting to analyse big data as part of strategic efforts to advance the nation’s health at the population level. This study explores the foundational goals, operational scope, future directions, and recommendations of the PHO in alignment with Vision 2030, considering its aim of enabling precision population health through artificial intelligence (AI)-enabled surveillance, equity-driven insights, and genomic integration. Drawing on global models, this review highlights PHOs’ role in population health data management, including data collection, analysis, and proactive predictive analysis to forecast diseases. It is intended to guide other entities and care providers towards prevention and provide them with the support needed to achieve this via data-driven insights, continuous follow-up, and impact assessment, along with the promotion of research and innovation. This should empower other healthcare branches and health policy translations. Implementation of the PHO faces significant challenges, including data sharing, fragmentation, quality, and digital infrastructure. However, the Saudi PHO also presents a significant opportunity to promote data sharing, research collaboration, and equitable distribution of healthcare resources. Integration of the PHO into the healthcare landscape is possible if we address these obstacles and take advantage of the available opportunities. The Saudi PHO is well placed to evolve into an entity that can shape and transform the Kingdom’s healthcare system while acting as a model for precision public/population healthcare and evidence-based healthcare-related decision-making across the Middle East and beyond.

## 1 Introduction

The Population Health Observatory (PHO), previously known as the Population Health Lab, was established in Saudi Arabia in 2023 with the support of Medical Services. It was intended to strengthen the Kingdom’s public health infrastructure through an improved data repository for disease burden monitoring and strategic policy guidance. Later, the PHO was moved to the remit of the Population Health Deputy under the auspices of the Ministry of Health (MoH). A decision was issued by His Excellency, the Minister of Health, Engineer Fahd Al-Jalajel, in December 2024 (1). In line with Vision 2030, the PHO addresses population-level health challenges and is poised to serve as a national platform for health intelligence while supporting precision public health (PPH) interventions. PPH refers to the use of high-quality population health data and analytics to deliver timely prevention, improve the effectiveness and equity of healthcare access, and ensure that appropriate interventions are targeted to the right group of people at the right time (2, 3). Inspired by international best practice model PHOs, such as the World Health Organization’s (WHO) Global Health Observatory (GHO), Public Health England (PHE), the European Health Information Initiative (EHII), the Centers for Disease Control and Prevention (CDC), Population Level Analysis and Community Estimates (PLACES), and the Behavioral Risk Factor Surveillance System (BRFSS), the Saudi PHO is intended to become a national hub for translating data into actionable insights (Table 1). These observatories worldwide have successfully demonstrated how structured data systems can support the real-time identification of at-risk populations, inform healthcare-related decision-making, and promote data equity (4–6).

**Table 1:** Comparison of the roles and responsibilities of national health observatories.

Observatory	Country/agency	Core functions	Data scope	AI and forecasting	Influencing policy
Population Health Observatory (PHO) (2, 3)	Saudi Arabia (MoH)	Disease surveillance, AI-enabled forecasting, policy translation, and monitoring health indicators and performance of all primary healthcare centres	National registries, EHRs, and genomic data	Moderate: Emerging integration	High: Alignment with Vision 2030
Global Health Observatory (GHO) (93)	World Health Organization (WHO)	Global health indicators, country comparisons, and Sustainable Development Goals monitoring	Multi-country, multi-source	Limited	High: Frameworks for global policy
PHE Knowledge & Intelligence Team (4)	United Kingdom (now UKHSA/O NS)	Local health profiles, inequality, and real-time dashboards	National health datasets, census data	Basic analytics	High: NHS and local government planning
EHII (European Health Information Initiative) (4, 17)	WHO Europe	Regional data harmonisation, knowledge sharing, and health reporting	Country-reported health statistics	Limited	Medium: Supporting European Union public health efforts
CDC PLACES and BRFSS (25)	United States	Behavioural risk factor surveillance, chronic disease mapping	Survey data, EHRs, social determinants	Moderate: geospatial models	Moderate to high

Globally, PHOs have evolved to consolidate health data as strategic platforms, monitor health equity, support evidence-based informed policymaking and integrate sensitive, real-time data with digital and analytical capabilities. International guidance has been placed, enabling cross-sectoral decision-making and strengthening health information systems (6–8). However, there have been few reports on evaluations of national-level PHOs in the Middle East aimed at examining their transformative effects on health systems and introducing how the PHOs operate via real-time monitoring to support population risk stratification as part of national policy (9).

The PHO currently operationalises PPH through integrated national data streams, stratified analytics and indicator-driven dashboards to identify at-risk individuals and geographical and demographic variations, thereby supporting targeted interventions, policymaking, and resource allocation. This review addresses this knowledge gap via the following primary objectives: (a) evaluating the introduction of the PHO as a transformative milestone in the development of Saudi Arabia’s healthcare system, (b) presenting the lessons learnt from its establishment as a national case study aligned with Vision 2030 and (c) evaluating the current role of the PHO and its future endeavours by comparing with globally relevant insights.

## 2 Methods

This manuscript presents a formative, descriptive implementation focused on the PHO establishment and early operationalisation, rather than a structured evaluation of the outcome benchmarking with international health observatory systems, looking into the world’s best practices in data-driven PHO. The international comparison is structured using predefined benchmarking domains such as the scope, governance, data integration, AI/forecasting capacity, and translation of the policy into preventive and interventive actions. To achieve this comparison, we used publicly available databases, including PubMed and Google Scholar, to investigate the mandates and outputs, providing a comparative benchmarking with international PHOs using predefined criteria such as data integration, governance, scope, and policy translation, in alignment with WHO guidance (10) on national health observatories.

## 3 Results

### 3.1 PHO establishment and rationale

The WHO has recommended that, given the need to systematically collect, disseminate, and analyse health information, countries should develop national health observatories (10). This need is particularly acute in Saudi Arabia because of regional disparities, fragmented data systems, and the rising burden of non-communicable diseases (11). A PHO can be particularly valuable in the face of these challenges by providing a holistic view of community health, considering other data besides those from the healthcare sector, including from spheres such as social services and education and variables such as social determinants of health (SDOH). It can also help bridge data silos, which is crucial for understanding health disparities (12, 13). In addition, a PHO can enable targeted interventions by enhancing analytics through continuous monitoring and evaluation to ensure the adequacy and effectiveness of the intervention (14, 15). A prompt collaborative framework is required for the obtained data to aid the development of evidence-based interventions tailored to specific populations (12, 14). Against this background, Saudi Arabia’s PHO was established using successful PHOs worldwide as models, particularly the one in the UK’s Office for Health Improvement and Disparities (OHID), which has validated the value of centralised health data systems (16, 17). Unfortunately, most international examples do not constitute proper technical validation but only demonstrate centralised data infrastructures. Thus, to support dissemination, the routine production and utilisation of health AI across diverse stakeholders should be operationalised. In this context, rather than simply testing system performance alone, PHOs within real-world healthcare systems must be validated in terms of functional utility, policy relevance, and governance integration (18). According to the WHO’s guidelines, national PHOs play important roles in empowering interoperability, standardised indicators, and appropriate decisions entrenched in national digital health strategies (19). This module of the national PHO exemplifies policy translation and strengthens the stewardship of the health system (9, 20, 21).

### 3.2 Current role and functions

#### 3.2.1 A national hub for population health data

**Figure 1:**
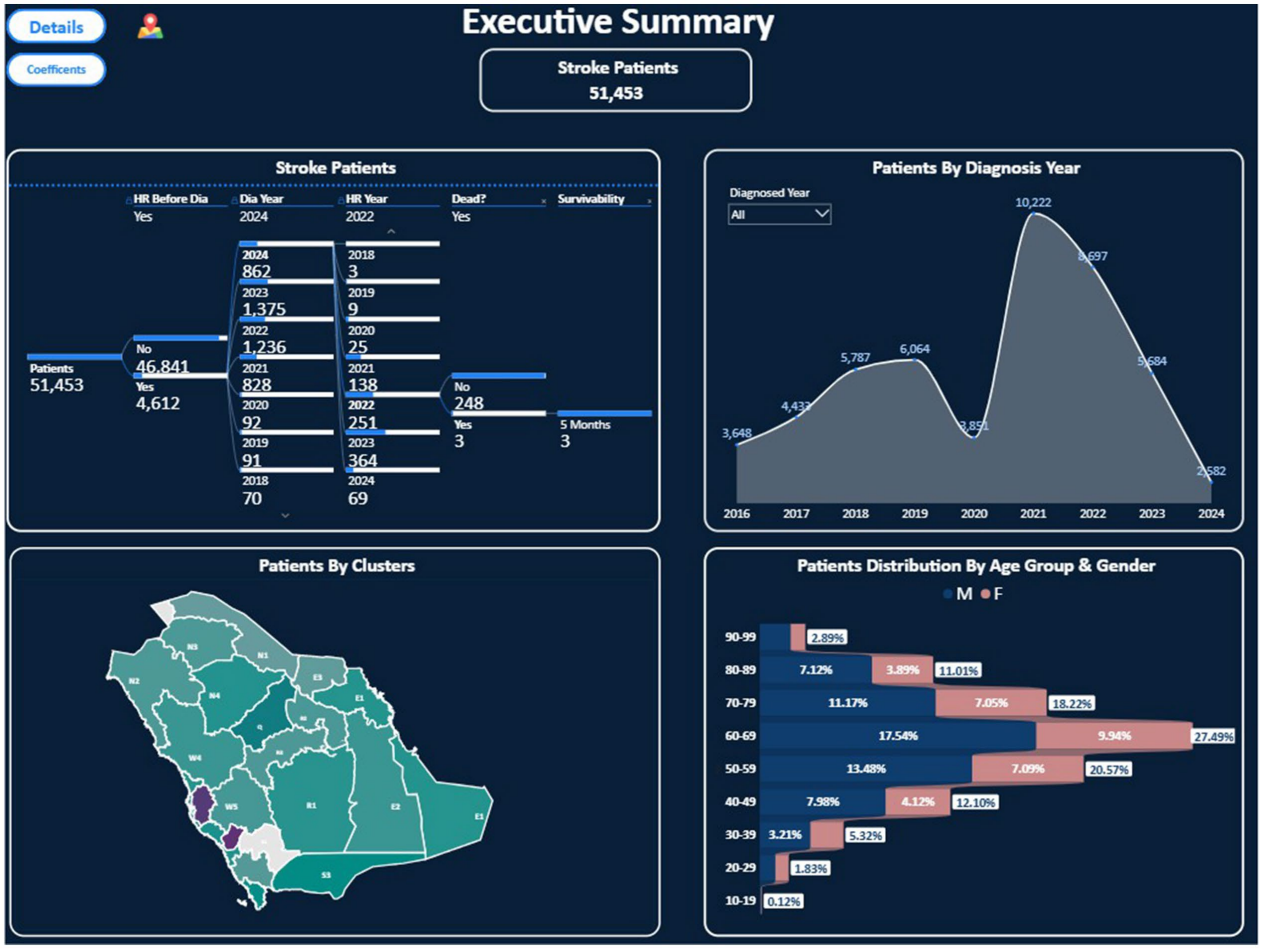
Executive summary of stroke patients in Saudi Arabia, presenting stroke patient numbers, patients by year of diagnosis, patients by cluster, and patient distribution by age and sex, adapted from the PHO at the MoH.

Through surveillance and reporting, Saudi Arabia’s PHO is currently responsible for tracking key public health indicators such as the prevalence of comorbidities and mortality rates, risk factors, and healthcare service utilisation rates (22, 23). This information is extracted and synthesised from multiple resources, such as electronic health records (EHRs), administrative databases, and national surveys (24). This standard was adapted from an international standard set by the WHO and the CDC (25, 26). As an example of such information compiled by the PHO, an executive summary of stroke patients in the Kingdom is shown in Figure 1. It indicates the number of cases and their location, sex, and age at diagnosis. This information is valuable for data-driven, precise decision-making, providing prompt intervention and early prevention where they are most required. This, in turn, helps ensure that the provided services are sustainable and of high quality in order to achieve better health outcomes.

#### 3.2.2 Burden estimation across geospatial mapping

To provide targeted interventions in high-risk or underserved communities, the PHO assesses disease distribution across regions by conducting a geospatial analysis (27, 28). This sheds light on regional health cluster performance and helps reduce regional health inequities (29).

#### 3.2.3 Data integration and policy evaluations

PHOs are also intended to be the main authoritative source of accurate and validated population-level health data. In the case of Saudi Arabia’s PHO, one of its main functions is to aid in the update of policies for national programmes, such as those against obesity and smoking and for promoting maternal health, as well as to evaluate their performance based on actual individual-level records rather than estimates (30–32). It also plays a critical role in integrating data across the MoH using demographic registries and Sehaty to support longitudinal analytics (29, 33, 34). Through this work, the PHO can help decision-makers by providing data-driven and evidence-based recommendations.

#### 3.2.4 Umbrella organisation supporting all health-related sectors

The sectors involved in health and healthcare-related legislative and/or medical activities in Saudi Arabia include the government, public, and private sectors. The role of the MoH is to oversee all healthcare providers, providing legislation and governance (35), while ensuring alignment across different sectors and engaging research centres, academic institutions, and regional public health departments (36). This collaboration also extends to international organisations, including the WHO, the Global Health Data Exchange (GHDx), and the World Bank, enhancing methodological transparency and benchmarking (18, 26, 28).

#### 3.2.5 Population health management

The PHO of Saudi Arabia’s MoH is also involved in stratifying health risks and priorities and introducing population health management (PHM) (2). This encompasses broader population-level strategies and prioritises individualised personalised care and stakeholder engagement to address changing demographics and disease patterns, health promotion, and policy development (37, 38). Integrated data analytics are crucial to enhance care delivery and understand the needs of the population in question, particularly when healthcare payment models change (39). Looking forward, the PHO is intended to expand into a national observatory of PPH, including data streams, to develop actionable insights from environmental, socio-economic, behavioural, and genetic perspectives (40–42). Collaboration with regional health clusters and universities will strengthen data governance capabilities and improve customised prediction models that are tailored specifically to Saudi Arabia’s epidemiological and demographic landscape (43, 44). Functioning within the PHO, the PHM should align with existing public health entities, such as the MoH regional health clusters, and the Public Health Authority requires clarification of analytical roles, data governance, and strategic responsibilities (29, 42). This aligns with Vision 2030 and should increase the average life expectancy and enable PPH (2, 45).

#### 3.2.6 AI integration and predictive analytics

The Saudi PHO currently utilises machine learning (ML) and AI for various healthcare-related purposes, which presents significant opportunities in epidemic modelling, hospital demand forecasting, and chronic disease risk prediction and stratification (46–48). Such models offer a substantial opportunity for analytics to support descriptive and stratified population health reporting through predictive dashboards and health behaviour trend models, rule-based risk stratification, data linkage across multiple sources, and automated generation of indicators to notify policy dashboards (49, 50). Furthermore, to improve public health responses, pattern recognition, such as natural language processing, could be used to enable disease spread forecasting and enhance epidemic preparedness (49, 50).

Current AI models primarily focus on enhancing analytic efficiency for decision-making support rather than on fully autonomous predictive modelling; however, they provide a foundation for the development of advanced AI applications. Benefits for primary healthcare and hospital demand forecasting can accrue from the PHO if it employs ML algorithms to analyse EHRs for predicting patient inflow and optimising hospital operations and resource allocation (51, 52). Predictive models can also improve triage and treatment planning by reducing costs and ensuring patient safety through predictive models (52). AI tools can also be used to stratify patients based on their likelihood of developing non-communicable diseases by identifying risk factors that can prevent their development or progression through targeted interventions (51, 53). These technologies can provide early warning alerts through predictive dashboards as well as health behaviour trend models, which could revolutionise proactive public health planning (49, 50). With scalability framed as future directions by a plan for phased developments, by integrating ML techniques for early warning systems, introducing population risk stratification with governance safeguards and validation processes. This aligns with evidence from around the world, emphasising that successful AI adoption in healthcare systems necessitates a development-staged approach relying on data transparency, maturity, human oversight, and robust data infrastructure (46–48).

### 3.3 International comparison

In 2013, the network of established PHOs in the United Kingdom was renamed Public Health England (PHE), which operates as a distinct entity. In 2021, it was reorganised into new national structures, performing public health intelligence and analysis. Since then, roles including monitoring of health inequalities, population health analytics, and policies lie within the remit of the Office for Health Improvement and Disparities (OHID), within which Knowledge and Intelligence Teams play critical roles nationally and regionally in evaluating the equity, quality, and outcomes of healthcare services (16). The UK Health Security Agency (UKHSA) is responsible for health protection intelligence. Consequently, PHOs, formerly existing as separate regional bodies, have been transformed into a centrally coordinated system embedded within national agencies, providing a more functionally distributed public health intelligence system (54, 55).

It publishes comprehensive reports guiding commissioning decisions and public health interventions on national health inequalities, preventable mortality, and healthcare accessibility (17). The Canadian Institute for Clinical Evaluative Sciences (ICES) redesigns health services, informs funding decisions, and provides best clinical practices. It integrates data from hospitals, registries, and community care to provide province-wide analyses (56). Similar practices are performed in the United States by the CDC, which maintains a robust health observatory infrastructure, such as the BRFSS. These practices include tracking health behaviours across all 50 states, which informs national prevention priorities (25). Furthermore, to help policymakers assess the impact of social determinants and prioritise community-level intervention tools, the County Health Rankings and Roadmaps Program has been used (57).

Outside of the developed world, the Ministry of Health and Family Welfare of India has created India’s National Health Observatory, which acts as a nationwide health observatory that serves as a central node for tracking service performance and delivery across the country’s decentralised healthcare systems (58). Moreover, in South America, Chile’s Health Intelligence System supports evidence-based planning and budgeting while monitoring population risk profiles and the performance of primary care networks (59).

Health intelligence infrastructures are rapidly shifting, addressing inefficiencies in population demographics and health system demands and improving healthcare outcomes (60, 61). The PHO combines various health data sources and advanced data integration techniques, enabling comprehensive surveillance beyond signal detection (62). This approach can enhance surveillance capabilities by incorporating social determinants of health (SDoH) that impact population health outcomes, particularly in response to national or global emergencies such as the COVID-19 pandemic (63).

## 4 Discussion

### 4.1 Key challenges and opportunities

This study reveals various challenges associated with the establishment of PHOs and the lessons learnt from this process. This section summarises the experiences at the early stage of PHO implementation and the benchmarking domains (as illustrated in Table 1), providing an analysis of how the project was implemented rather than an evaluation based on its outcomes. Such an analysis of implementation involves examining structured governance and implementation records, formative synthesis of programme documentation, data-integration milestones, indicator development workflows, and policy briefing outputs. Furthermore, during its establishment, internal iterative review meetings and early operational phases of the PHO were held.

There are several crucial challenges to overcome in order to successfully integrate a PHO. One major obstacle is the fragmentation of health data. By nature, health data are disjointed, making it difficult to obtain a cohesive overview of a patient and limiting the interoperability. This can lead to suboptimal and inefficient care delivery (64). Ethical handling of data and the protection of data privacy must be assured, particularly for health data that can include patients’ personal information or clinical, genomic, and psychological findings. In this context, adequate control of data sharing is essential to comply with privacy regulations and maintain trust (65, 66). At the institutional level, ambiguous legal frameworks can hinder operational effectiveness and authority (67). Moreover, the existence of multiple health departments and entities at the national level might lead to confusion regarding who holds the roles and responsibilities, which might dilute the impact of health observatories (9).

### 4.2 Limitations and implementation challenges of PHOs

To establish effective PHOs, inherent challenges must be overcome, including data-related limitations such as fragmented datasets, low-quality data (e.g., misclassification, missingness, and inconsistency of denominators), and limited data availability. Governance limitations, which include privacy safeguards, equity, and inconsistent standards, are associated with completeness and linkage, along with interoperability barriers such as timeliness, responsiveness of intelligence products, long refresh cycles, and delayed reporting (38, 68–70).

Given the use of complex and sensitive data, the effective implementation of a PHO requires a skilled workforce, which can be a bottleneck given the notable lack of professionals with expertise in data quality, health data policy, bioinformatics, and data science (71). With skilled staff, classification issues arise when many professionals lack classifications, leading to demotivation that affects their engagement and productivity (72). These challenges and opportunities associated with PHO operational functions, which are described in Section 3.2, include surveillance reporting surveys, dashboards with geospatial mapping, evaluation of current policies, and AI-enabled analytics. Further contextualised through the international benchmarking of the PHO summary (Table 1).

Despite the challenges associated with the implementation of a PHO, these are outweighed by opportunities to enhance the utilisation of health-related and other data to promote public health while fostering partnerships between AI researchers, academics, and healthcare professionals (73). Moreover, by integrating data from diverse sources, the PHO can act as an engine to support policymaking that addresses the social determinants of health, ultimately improving health outcomes (Kechadi, 2016). Additionally, the PHO’s effects of promoting health equity throughout Saudi Arabia can ensure that healthcare resources are allocated fairly across the different regions of the country (71). Considering the future, the PHO still has several milestones to pass along the path to achieve its full potential. These future endeavours (as described in the subsections below) were considered consequential based on planning discussions and a review of the MoH’s transformation objectives, which were further augmented by comparisons with the remit and functions of PHOs internationally (e.g., WHO GHO, UK OHID, ICES, and CDC frameworks), as described in this manuscript.

#### 4.2.1 Institutional development and autonomy

The PHO has the potential to grow into a *national public health intelligence* with links to academia, enabling innovation and independent evaluation (74). However, this requires a legal framework to ensure that data sharing occurs appropriately and safely (75).

#### 4.2.2 Building capacity

It is necessary to develop collaborative programmes between national and international public health agencies and universities to create a skilled workforce capable of analysing and interpreting different sets of data (34, 76).

#### 4.2.3 Public communication

Knowledge translation is an essential role of the PHO, which should involve the regular issuance of publications such as risk reports and health bulletins, along with dashboards to provide real-time visual updates, not only for policymakers but also for the public through the Digital Twin (DT), healthcare professionals, and the media. This can also provide data-driven evidence translation, which is key to maximising data utility (18, 75, 77).

#### 4.2.4 Innovation through research and development

The PHO provides real-time updates on the disease burden and SDoH on a national scale, focusing on those with disabilities and other vulnerable populations, including migrants, by monitoring, updating, and creating solutions for new and impactful national policies (78, 79). For future PHO development, the integration of indicators reflecting commercial and digital determinants, including environmental exposure, consumption of unhealthy products, and utilisation patterns, should also be considered while strengthening digital transformation efforts and monitoring health equities with a focus on reducing disparities (80, 81).

#### 4.2.5 Genomic and biological data

Rather than focusing solely on individuals, genetic data can be monitored across populations to prevent diseases and promote health (82). Screening programmes can also be monitored and updated, accelerating the identification of rare diseases and providing early interventions, thus facilitating personalised medical plans that can improve public health outcomes (83). The PHO can also monitor current programmes for screening newborns and couples prior to attempts to conceive, including efforts to prevent rare diseases (84–87). It can also provide a database that accelerates the identification of genetic variations arising from the founder effect, which is associated with the Saudi population, and recalibrates variants of unknown significance (88, 89). Models for predicting diseases with complex inheritance can also be employed to identify the possibility of psychological, behavioural, and other complex phenotypes based on genetic/genomic sequences (77, 90). Beyond genomics, the integration of multi-omics technologies (i.e., metabolomics, proteomics, transcriptomics and epigenetics) will provide a more holistic view of health, incorporating the accurate prediction of biological functional modules and enabling comprehensive health assessments that correlate with health conditions (91, 92). This will activate the DT with a precision model, such as the BFM-ash model, which provides an assessment of health status based on biological functional modules (92). This PPH approach within the PHO is reflected in (i) the development of standardised indicators, (ii) datasets integrating data from multiple sectors, (iii) interactive dashboards to aid decision-making, and (iv) reporting stratified by individual or region, and alternatively nationwide, to support prioritisation and targeted interventions.

Despite the substantial efforts made by the MoH to implement the Saudi PHO, which oversees the country’s healthcare system and its activities, clear legal autonomy aligned with the national health strategy is required. There is also a need to introduce a performance index to provide accurate assessments for decision-makers.

## 5 Conclusion

The PHO stands at the forefront of Saudi Arabia’s digital health transformation and is poised to become a powerful engine for policy reform, assessing decision-making, and proactive healthcare delivery, making it a cornerstone of Saudi Arabia’s health intelligence. This observatory has the potential not only to evolve into a regional model for passive data aggregation in the form of EHRs and biological, genomic, and social determinants of health and disease but also to adopt a strategic role as an equity-driven, evidence-based public health body. Aligned with Saudi Arabia’s Vision 2030, the PHO introduced international best practices, placing it at the forefront of regional innovation. To unlock its full potential, the PHO must prioritise knowledge translation by gathering further datasets, investing in predictive analytics, and pursuing institutional autonomy.
