# Correction: The role and future of the population health observatory: advancing public health intelligence in Saudi Arabia

**DOI:** 10.3389/fpubh.2026.1818017

**Published:** 2026-03-17

**Authors:** Mariam M. Al Eissa, Malak E. Aloufi, Abdulaziz Eskandarani, Aisha M. Alshehri, Mohammed M. Alrwois, Yahya A. AlMazni, Shaker A. Alomary, Abdullah Assiri, Mohammed AlAbdulaali

**Affiliations:** 1 College of Medicine, Alfaisal University, Riyadh, Saudi Arabia; 2 Population Health Observatory, Ministry of Health, Riyadh, Saudi Arabia; 3 Public Health Lab, Public Health Authority, Riyadh, Saudi Arabia; 4 King Khaled Eye Specialist Hospital (KKESH) and Research Centre, Riyadh, Saudi Arabia; 5 Computational Sciences Department at the Centre for Genomic Medicine (CGM), King Faisal Specialist Hospital and Research Center, Riyadh, Saudi Arabia

**Keywords:** population health observatory, public health intelligence, health equity, AI in healthcare, vision 2030

Affiliation “College of Medicine, Alfaisal University, Riyadh, Saudi Arabia” was omitted for author Abdullah Assiri.

This affiliation has now been added for author Abdullah Assiri.

In the published article, there was an error in the order of affiliations. The correct order of affiliations is listed below.

Mariam M. Al Eissa^1, 2, 3, 4, 5*^, Malak E. Aloufi^2^, Abdulaziz Eskandarani^2^, Aisha M. Alshehri^2^, Mohammed M. Alrwois^2^, Yahya A. AlMazni^2^, Shaker A. Alomary^2^, Abdullah Assiri^1, 2^ and Mohammed Abdulali^2^

^1^College of Medicine, Alfaisal University, Riyadh, Saudi Arabia.

^2^Population Health Observatory, Ministry of Health, Riyadh, Saudi Arabia.

^3^Public Health Lab, Public Health Authority, Riyadh, Saudi Arabia.

^4^King Khaled Eye Specialist Hospital (KKESH) Research Centre, Riyadh, Saudi Arabia.

^5^Computational Sciences Department at the Centre for Genomic Medicine (CGM), King Faisal Specialist Hospital and Research Center, Riyadh, Saudi Arabia.

The original version of this article has been updated.

